# The Mechanoreception in *Drosophila melanogaster* Oocyte under Modeling Micro- and Hypergravity

**DOI:** 10.3390/cells12141819

**Published:** 2023-07-10

**Authors:** Irina V. Ogneva

**Affiliations:** 1 Cell Biophysics Laboratory, State Scientific Center of the Russian Federation Institute of Biomedical Problems of the Russian Academy of Sciences, 76a, Khoroshevskoyoe Shosse, 123007 Moscow, Russia; iogneva@yandex.ru or iogneva@imbp.ru; Tel.: +7-(499)195-63-98; 2 Medical and Biological Physics Department, I. M. Sechenov First Moscow State Medical University, 8-2 Trubetskaya Street, 119991 Moscow, Russia

**Keywords:** cell mechanosensitivity, oocyte, cytoskeleton, cell stiffness, cellular respiration, weightlessness

## Abstract

The hypothesis about the role of the cortical cytoskeleton as the primary mechanosensor was tested. *Drosophila melanogaster* oocytes were exposed to simulated microgravity (by 3D clinorotation in random directions with 4 rotations per minute—sµg group) and hypergravity at the 2 g level (by centrifugal force from one axis rotation—hg group) for 30, 90, and 210 min without and with cytochalasin B, colchicine, acrylamide, and calyculin A. Cell stiffness was measured by atomic force microscopy, protein content in the membrane and cytoplasmic fractions by Western blotting, and cellular respiration by polarography. The obtained results indicate that the stiffness of the cortical cytoskeleton of *Drosophila melanogaster* oocytes decreases in simulated micro- (after 90 min) and hypergravity (after 30 min), possibly due to intermediate filaments. The cell stiffness recovered after 210 min in the hg group, but intact microtubules were required for this. Already after 30 min of exposure to sµg, the cross-sectional area of oocytes decreased, which indicates deformation, and the singed protein, which organizes microfilaments into longitudinal bundles, diffused from the cortical cytoskeleton into the cytoplasm. Under hg, after 30 min, the cross-sectional area of the oocytes increased, and the proteins that organize filament networks, alpha-actinin and spectrin, diffused from the cortical cytoskeleton.

## 1. Introduction

The influence of physical fields on a cell, in contrast to various chemical stimuli, is one of the least studied issues in cell biology. Of particular difficulty is the study of the interaction between the cell and the gravitational field. However, the results of such studies are absolutely necessary for the development of new methods for protecting the human body from the negative impact of microgravity, which occurs in long-term space flights, especially in deep space exploration.

The cell was formed and evolved under conditions of a constant gravitational field. In other words, the change in gravity was not presented as a stimulus to the cell in its natural evolution. Considering the change in gravity as a mechanical effect, it can be assumed that the act of primary reception is the occurrence of deformation of the cellular structure [1,2]. Indeed, in the “FLUMIAS” space experiment, direct experimental evidence was obtained: in suborbital flight, the shape of the cells changed almost immediately after the onset of weightlessness [3].

The shape of cells is determined primarily by the cytoskeleton. In real and simulated micro- (2D or 3D clinorotation by random position machine) and hypergravity (by centrifugation), a change in the structure of all types of filaments involved in the formation of the cytoskeleton was shown, and experiments were performed on cells of various types and ls of differentiation [4,5,6,7,8,9,10].

In addition, under gravity change, a change in various links of cellular metabolism is observed. In the space experiment “TRIPLELUXA”, in the NR8383 line of rat alveolar macrophages, the oxidative burst stopped immediately upon reaching weightlessness but was restored after 42 s [11]. Also, under microgravity conditions, cellular respiration (oxidative phosphorylation (OXPHOS)) changes in various types of cells, both in tissues and in cultured ones [12,13,14]. Moreover, such changes in cellular metabolism can be mediated, among other things, by changes in the structure of the cytoskeleton [15,16].

However, despite the large amount of experimental data indicating various structural and functional changes in cells with a change in gravity, there is still no unequivocal answer as to which structure acts as a receptor for changes in the gravitational field. Summarizing the above, in many works, there is an indication of a change in the structure of the cytoskeleton with the force of gravity changes [2] and even proposed to consider it as a whole as a sensor [17]. In addition, the cytoskeleton affects metabolic homeostasis and the production of macroergs necessary to maintain cell life [16]. However, in the evolutionary series, not all cells have a developed network of filaments of different types that penetrate the entire cell. But all cells have a cortical cytoskeleton that supports the cell membrane—the boundary between the cell and the environment—and that, in fact, determines the shape of cells. Therefore, it was previously proposed to consider the cortical cytoskeleton as a primary mechanosensor, the various deformations of which (depending on the increase or decrease in external mechanical action) lead to the launch of the corresponding signaling pathways and the formation of an adaptive structural and functional pattern [1,2]. Moreover, an increase in the stiffness of the cortical cytoskeleton increases resistance to deformation and reduces the response to gravity changes [18].

The aim of this work was to experimentally test the hypothesis about the role of the cortical cytoskeleton in the mechanoreception of *Drosophila melanogaster* oocytes. The choice of this object is due to several reasons. The main reason is that dechorionized *Drosophila* oocytes can be exposed, for example, on agar plates in a humid atmosphere to avoid drying out, but not in liquid, which eliminates artifacts associated with fluid shift (fluid flow can induce shear stresses in cultured cells). In addition, our previous results indicated that oocytes collected from flies immediately after a space flight have structural changes that are reflected in changes in the mechanical characteristics of the cells [19]. Finally, the study of the response of mature oocytes to changes in gravity is important in the search for effective measures to protect female reproductive health during and after space flights.

*Drosophila melanogaster* oocytes were exposed for 30, 90, and 210 min in simulated microgravity (by 3D clinorotation) and hypergravity at 2 g (by centrifugal force from one axis rotation). The maximum cross-sectional area of oocytes was measured as a marker of the onset of deformation and the stiffness of the cortical cytoskeleton for an integral assessment of the structure, including using various agents for the selective disassembly of microfilaments (2 µM cytochalasin B), microtubules (10 µM colchicine), intermediate filaments (40 mM acrylamide), and for cortical actin condensation (100 nM calyculin A). In addition, the content of cytoskeletal proteins in the membrane and cytoplasmic fractions of proteins was determined, and the rate of cellular respiration was evaluated as an indicator of the functional state of oocytes.

## 2. Materials and Methods

### 2.1. Experimental Design

Virgin 5-day-old female fruit flies *Drosophila melanogaster* were placed into cages with agar plates, prepared according to [20], to collect oocytes. The harvested oocytes were dechorionized with sodium hypochlorite (as a 50% bleach) according to the protocol [20].

The oocytes were then transferred to insect saline, which, depending on the series of experiments, either did not contain any drugs or contained 2 µM cytochalasin B (for disrupting microfilaments), 10 µM colchicine (for disrupting microtubules), 40 mM acrylamide (for disrupting intermediate filaments) [21], or 100 nM calyculin A (for cortical actin condensation) [22,23]. Pre-incubation was carried out for 3 h. All manipulations with oocytes were performed at room temperature.

After pre-incubation, oocytes were collected and transferred to wet agar plates, which were randomly divided into experimental and control groups. Experimental dishes were placed on the Gravite platform (Gravite^®^, GC-US-RCE010001, Space Bio-Laboratories Co., Ltd., Hiroshima, Japan) to simulate microgravity (sµg group) or hypergravity at 2 g (hg group) for 30, 90, and 210 min. In accordance with the manufacture’s manual, Gravite^®^ is a multi-directional gravity device for simulating microgravity and hypergravity. By controlled rotation of the two axes, the 3D clinostat minimizes the cumulative gravity vector at the center of the device and makes 10^−3^ g over time average. This method is generally accepted for simulating weightlessness on Earth, including for *Drosophila melanogaster* [24], and this device has been used in many studies on cultured cells [25,26,27]. Mode A was used in this study: 4 rotations per minute with random directions, so simulated weightlessness was achieved in average every 15 s. Gravite^®^ can also create a hypergravity environment at the 2 g or 3 g level by centrifugal force from one axis rotation. Each experimental group corresponded to a control group of oocytes (group C) placed on another dish, which was under the same environmental conditions as the experimental one.

Each study group had at least 100 oocytes. All experiments were repeated at least three times.

All experimental procedures were approved by the Commission on Biomedical Ethics of the Institute of Biomedical Problems (IBMP), the State Scientific Center of the Russian Federation (Minutes No. 624 dated 20 October 2022).

### 2.2. Light Microscopy

After exposure under simulating microgravity or hypergravity conditions, control and experimental oocytes were transferred from agar plates and placed on the glass. Images were acquired using an IX73 inverted microscope (Olympus Corporation, Tokyo, Japan) in transmitted light at 20× magnification. The focus during image acquisition was chosen so that the area of the oocyte was maximized. At least 100 cells were used for staining for each group. The results (minimal and maximum linear sizes and the area) were analyzed using the Fiji package (https://imagej.net/Fiji, accessed date 2 May 2023).

### 2.3. Atomic Force Microscopy

The technique for measuring the stiffness of *Drosophila melanogaster* oocytes was described in detail earlier [19]. Briefly, an NTEGRA NEXT II atomic force microscope (NT-MDT, Moscow, Russia) with a soft silicon cantilever (CS17, NT-MDT, Moscow, Russia) was used for measurement. The intrinsic stiffness of the cantilever was 69 pN/nm, according to the determined resonant frequency of its natural oscillations. Dechorionized oocytes after the end of exposure or the control group were placed on a rigid substrate, and force curves were obtained in the contact mode. The calibration curve was recorded on a substrate outside the oocytes to recalculate the measured parameters into the actual indentation depth and the applied force. The stiffness (in pN/nm) was calculated as the ratio of the applied force (in N) to the indentation depth (in m), which, as described before, was 50 nm.

### 2.4. Western Blotting

After the end of exposure under conditions of simulated microgravity or hypergravity, oocytes from the experimental and control groups were collected from agar plates, lysed, and the membrane and cytoplasmic protein fractions were isolated according to [28]. For electrophoresis, the same amount of protein was applied to each well based on concentration measurements. After electrophoresis in SDS-polyacrylamide gel, the proteins were transferred onto nitrocellulose membranes. The loading control and transfer efficiency were assessed using Ponceau staining. The membranes were then washed in PBST, blocked in 4% skim milk powder, and stained with specific primary antibodies at dilutions recommended by the manufacturers (Table 1). HRP-linked horse and goat antibodies for chemiluminescent detection were used as secondary antibodies to detect rat IgG, mouse IgG, and rabbit IgG (#7077S, #7076S, and #7074S, accordingly, Cell Signaling Technology, Inc., Danvers, MA, USA) at a dilution of 1:10,000. The membranes were then treated with SuperSignal™ West Femto Maximum Sensitivity Substrate (#34,096, Thermo Scientific™, Waltham, MA, USA) at a dilution of 1:10. The protein bands were revealed using the ChemiDoc XR+ System and processed using Image Lab Software (Bio-Rad Laboratories, Hercules, CA, USA).

### 2.5. Polarography

In this series of experiments, oocytes from the control groups and after exposure were collected from the agar plates, counted, and placed in saline for insects. Next, the oocytes were permeabilized for 10 min with 10 µg/mL saponin and a substrate-inhibitor analysis was performed according to the protocol of Kuznetsov A.V. et al. [29] in the polarographic cuvette of the Oxygraph+ instrument (Hansatech Instruments Ltd., King’s Lynn, Norfolk, UK), as described in detail earlier [14]. All procedures were carried out at room temperature.

We measured the basal oxygen uptake rate V0, the uptake rate with the addition of a mixture of glutamate and malate (10 mM glutamate + 5 mM malate), Vglu + mal, and the maximum respiratory rate with the addition of 2 mM ADP, Vmax. Then, inhibitors and substrates of the following complexes of the respiratory chain were added in turn: 0.5 µM rotenone (complex I inhibitor), 10 mM succinate (substrate of complex II)—the rate of oxygen uptake V (II) was recorded—5 µM antimycin A (complex III inhibitor), and 0.5 mM TMPD + 2 mM ascorbate (artificial substrates of complex IV)—recorded the oxygen uptake rate V (IV). After that, for each sample, a test was performed for the integrity of the outer mitochondrial membrane by adding 10 μM cytochrome c. The rate of cellular respiration was expressed in pmol O2 per ml per min per oocyte.

### 2.6. Statistical Analysis

The analysis of the obtained results was carried out using a two-way analysis of variance (two-way ANOVA) using Student’s posterior t-test with Bonferroni’s correction for multiple comparisons. A significance level of *p* < 0.05 was used to assess the significance of changes. Data are presented as M ± SE (M is the arithmetic mean, SE is the standard error of the mean).

To obtain each average, the results from a minimum of three biological replicates were used. When measuring size and stiffness, at least 30 oocytes in each biological replica were tested.

## 3. Results

In all studies, each measurement point had its own control point to avoid artifacts associated with the exposure time.

### 3.1. Linear Sizes and Area of Maximum cross-Section of Oocytes

Cell deformation manifests itself in a change in their shape, which was assessed using the linear sizes and area of the maximum cross-section of oocytes.

The linear sizes and area of the maximum cross-section of oocytes in the control groups did not differ over time.

**Figure 1:**
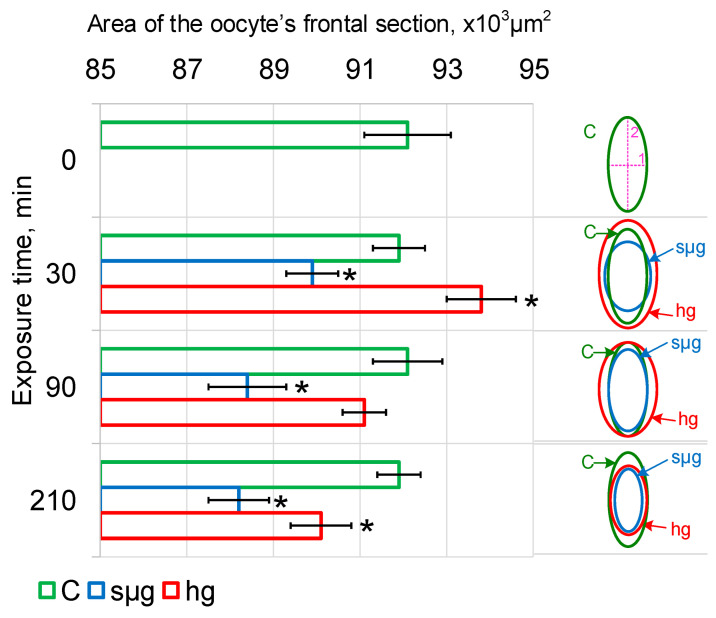
Dynamics of the area of maximum cross-section of *Drosophila melanogaster* oocytes during exposure under simulated micro- and hypergravity conditions: C, control (green); sµg, simulated microgravity (blue); and hg, hypergravity at the 2 g level (red). The scheme of oocyte deformation in the dynamics of simulated micro- and hypergravity is presented in the right panel. Purple lines mark the axes of the oocyte as an ellipse: 1, minor axis; and 2, major axis. The dynamics of changes in the lengths of the minor and major axes are shown in Table 2. * *p* < 0.05 in comparison with the corresponding control group.

Under simulated microgravity, after 30 min the minimum linear size (purple line 1 on the right panel Figure 1) increased by an average of 3 µm (*p* < 0.05), after 90 min it decreased to the control level, and after 210 min it was lower than the control by an average of 2.5 µm (*p* < 0.05). At the same time, the maximum linear size (purple line 2) decreased by an average of 7.2 µm (*p* < 0.05) after 30 min and remained reduced up to 210 min of exposure (Table 2). Accordingly, the maximum cross-sectional area of *Drosophila melanogaster* oocytes decreased already after 30 min and then remained reduced up to 210 min of exposure in simulated microgravity (Figure 1).

**Figure 2:**
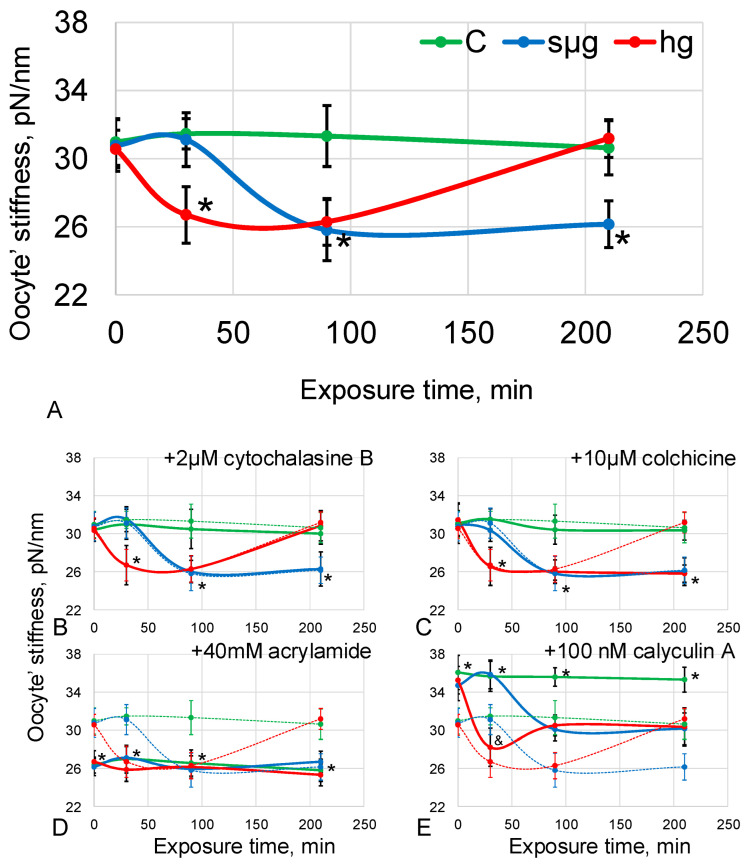
Dynamics of stiffness of *Drosophila melanogaster* oocytes during exposure to simulated micro- and hypergravity. The following is as described above: C, control (green); sµg, simulated microgravity (blue); and hg, hypergravity at the 2 g level (red). On all panels, the axes are the same as on panel (**A**): the *y*-axis is the oocyte’s stiffness, pN/nm; the *x*-axis is the exposure time, min. (**A**) Pre-incubation without any agents. (**B**) Pre-incubation with 2 µM cytochalasine B (disrupt microfilaments). (**C**) Pre-incubation with 10 µM colchicine (disrupt microtubules). (**D**) Pre-incubation with 40 mM acrylamide (disrupt intermediate filaments). (**E**) Pre-incubation with 100 nM calyculin A (cortical actin condensation). On panels (**B**–**E**), the values of panel A are plotted with dashed lines of the corresponding color. In some cases, especially in panels (**B**,**C**), the values are very close and the dotted lines overlap with the main plot. * *p* < 0.05 in comparison with the according control group without any agents, and *p* < 0.1 in comparison with the according control group without any agents.

Under hypergravity conditions, the minimum linear size (purple line 1 on the right panel of Figure 1) after 30 and 90 min exceeded the control by an average of 5 µm (*p* < 0.05) (Table 2) but returned to the control level by 210 min. The maximum linear size (purple line 2) after 30 min was larger than that of the control by an average of 5.7 μm (*p* < 0.05); after 90 min there was a downward trend (*p* < 0.1), which led to a significant decrease after 210 min by an average of 7.4 µm (*p* < 0.05). The area of the maximum section of oocytes under hypergravity conditions after 30 min was greater than that in the control (*p* < 0.05), after 90 min it did not differ from the control, and after 210 min it was lower than that in the control (*p* < 0.05) (Figure 2).

The obtained results indicate that there was a change in the shape of *Drosophila melanogaster* oocytes after 30 min, both under conditions of simulated microgravity and hypergravity at the level of 2 g. Since the shape of the cells is determined primarily by the cytoskeleton, for the integral assessment of the structure, the measurement of stiffness by atomic force microscopy was used.

### 3.2. Oocyte’ Stiffness

The results obtained showed that the stiffness of oocytes in the control groups without or with the addition of disrupting agents did not differ over time in each block of the study.

In addition, to avoid artifacts associated with a 3 h incubation of oocytes in insect saline, either without or with drugs that cause disassembly of cytoskeletal components, stiffness measurements were performed under simulated micro- and hypergravity conditions without prior incubation. The results obtained without incubation and with 3 h incubation in insect saline without the addition of disrupting agents were not different (Table S1).

Oocyte stiffness under simulated microgravity decreased by 16% (*p* < 0.05) after 90 min and remained at the same level by 210 min of exposure. Under hypergravity conditions, stiffness decreased by 15% after 30 min (*p* < 0.05), remained at the same level after 90 min, and returned to the control level after 210 min (Table S1, Figure 2A). 

A decrease in stiffness indicates a change in the structure of the cortical cytoskeleton under simulated micro- and hypergravity. Since the cytoskeleton is multicomponent, it remains unclear which structures cause a decrease in stiffness with changes in external mechanical conditions.

Pre-incubation with 2 µM cytochalasin B (disrupting microfilaments) did not change the stiffness relative to the groups without the addition of disrupting agents. Moreover, the dynamics under simulated micro- and hypergravity conditions were the same as those described above (Table S1, Figure 2B). This indicates that at simulated 0 g and 2 g, microfilaments of *Drosophila melanogaster* oocytes are not involved in the change in stiffness; otherwise, the dynamics of stiffness change would change.

Pre-incubation with 10 µM colchicine (disrupting microtubules), as well as with the addition of cytochalasin B, did not change the stiffness relative to groups without the addition of disrupting agents. However, the dynamics differed; the same decrease was noted at the same time as without disrupting agents (after 90 min for sµg by 17% (*p* < 0.05); after 30 min for hg by 16% (*p* < 0.05)), but after 210 min, stiffness in the hg group did not recover (Table S1, Figure 2C). As with microfilaments, *Drosophila melanogaster* oocyte microtubules are not involved in stiffness reduction in simulated 0 g and 2 g but are required for its restoration.

Pre-incubation with 40 mM acrylamide (disrupting intermediate filaments) resulted in a 16% reduction in stiffness (*p* < 0.05) in the control group compared to the control group without disrupting agents. At the same time, exposure to simulated microgravity, as well as to hypergravity, did not lead to a change in stiffness relative to the control group; it was still reduced by 15–17% (*p* < 0.05) (Table S1, Figure 2D). The decrease in stiffness in the control and the absence of subsequent changes during exposure to 0 g and 2 g suggests that the intermediate filaments were involved in the observed decrease in stiffness with changes in external mechanical conditions.

If deformation leads to a change in the structure of the cortical cytoskeleton, then an increase in its stiffness prior to exposure may level the changes. In the absence of specific agents to condense intermediate filaments in the cortical cytoskeleton, calyculin was used to condense actin filaments.

Pre-incubation with 100 nM calyculin A (cortical actin condensation) resulted in an 18% increase in oocyte stiffness (*p* < 0.05) compared to the group without agents added. After 90 min of simulated microgravity, oocyte stiffness decreased by 13% (*p* < 0.05) compared with the corresponding control group and did not differ from the level of the control without the addition of agents. After 210 min of simulated microgravity, the stiffness was the same as after 90 min. Under 2 g conditions, oocyte stiffness was lower by 20% (*p* < 0.05) relative to the corresponding control and, moreover, tended to decrease (by 9%, *p* < 0.1) relative to the control group without the addition of agents. However, after 90 min, the stiffness increased slightly and did not differ further from the level of the control group without the addition of agents (Table S1, Figure 2E). The data obtained indicate that an increase in the stiffness of the cortical cytoskeleton prior to exposure reduces sensitivity to changes in the external mechanical field, but the dynamics of changes emphasizes that microfilaments are not responsible for the decrease in stiffness.

### 3.3. Cytoskeletal Proteins’ Content in the Oocytes

In addition to using disrupting agents to determine the role of cytoskeletal components in reducing the stiffness of *Drosophila melanogaster* oocytes under conditions of simulated micro- and hypergravity, the contents of the main proteins that form cytoskeletal structures were determined. In addition, the key question remained: how exactly, at the molecular level, is different deformation realized in the formation of a different (for micro- and hypergravity) adaptive pattern?

For each protein, in the membrane and cytoplasmic fractions of proteins, the content in the control groups at each time point (after 30, 90, and 210 min) did not significantly differ from the control at the beginning of exposure (0 min).

**Figure 3:**
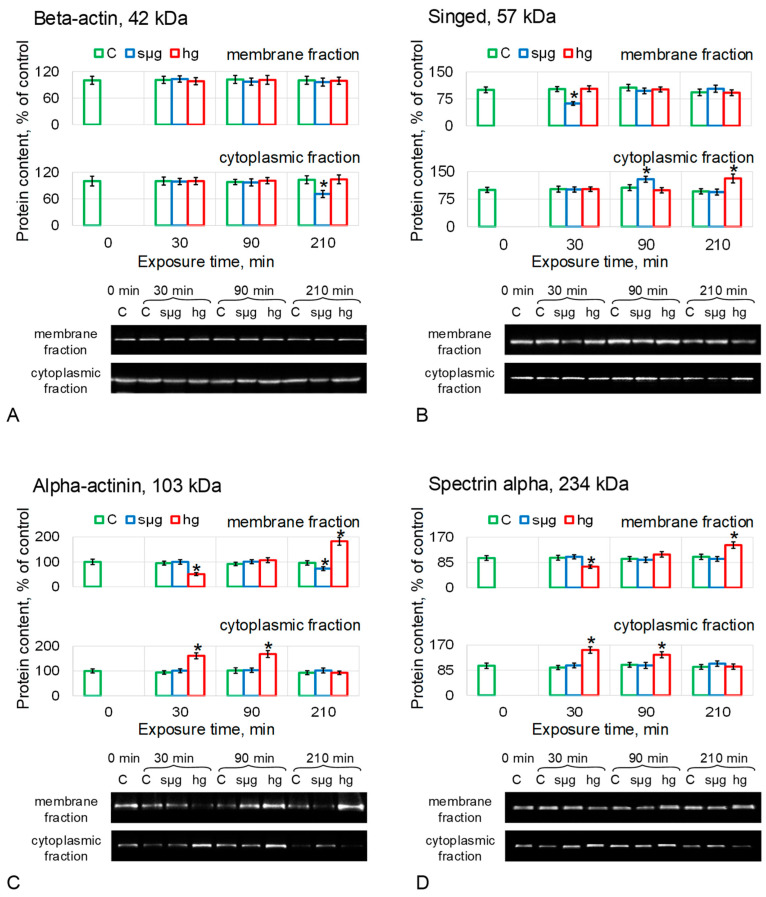
Relative protein content (microfilament proteins) in the membrane and cytoplasmic fractions of *Drosophila melanogaster* oocytes during exposure to simulated micro- and hypergravity. As described above: C, control (green); sµg, simulated microgravity (blue); and hg, hypergravity at the 2 g level (red). Each panel presents typical Western blot (for clarity) and histograms of changes in the content of proteins in the membrane and cytoplasmic fractions as a percentage of the corresponding control at each time point. * *p* < 0.05 in comparison with the corresponding control group. (**A**) Beta-actin, Actb. (**B**) Singed, Sn (fascin homologue), actin binding protein, formed bundles. (**C**) Alpha-actinin, Actn, actin binding protein, formed network. (**D**) Spectrin alpha, AlphaSpec, formed network.

The relative content of beta-actin in the membrane fraction (MF) of proteins did not change in any of the groups (Figure 3A). The cytoplasmic fraction (CF) also showed no change other than a 29% decrease (*p* < 0.05) in the simulated microgravity group after 210 min of exposure (Figure 3A).

The content of Sn in MF under simulated microgravity dropped by 38% (*p* < 0.05) after 30 min of exposure (Figure 3B), but after 90 min, it recovered to the control level and did not change further. At the same time, in CF it increased by 29% after 90 min (*p* < 0.05), but after 210 min, it did not differ from the control. In the hg group, the Sn content in MF did not change; in CF, it increased by 31% (*p* < 0.05) after 210 min (Figure 3B).

Actn content in MF in the sµg group decreased by 27% after 210 min (*p* < 0.05) but remained unchanged in CF (Figure 3C). In the hg group, after 30 min of exposure, the Actn content in MF decreased by 49% (*p* < 0.05) and increased in CF by 61% (*p* < 0.05). After 90 min, the Actn content in MF was restored to the control but remained higher by 68% (*p* < 0.05) in CF. After 210 min, the Actn content in MF oocytes of the hg group was 82% higher than the control (*p* < 0.05), but in CF, it did not differ from the control (Figure 3C).

The relative content of AlphaSpec did not change in MF and CF of *Drosophila melanogaster* oocytes under simulated microgravity (Figure 3D). In the hg group, the dynamics of AlphaSpec content was similar to Actn; in MF, after 30 min, the content of AlphaSpec decreased by 29% (*p* < 0.05) but increased in CF by 53% (*p* < 0.05). After 90 min in MF, it did not differ from the control; in CF, it was higher by 37% (*p* < 0.05). After 210 min, the relative content of AlphaSpec in MF exceeded the control by 43% (*p* < 0.05), and in CF, it did not differ from the control (Figure 3D).

The results of determining the content of proteins forming microfilament structures showed that actin remained intact (which is consistent with stiffness measurements, including the use of the disrupting agent cytochalasin). Under conditions of simulated microgravity, the content of the singed protein, which forms bundles, changed. Under conditions of hypergravity, the content of proteins that organize networks, alpha-actinin and spectrin alpha, changed.

**Figure 4:**
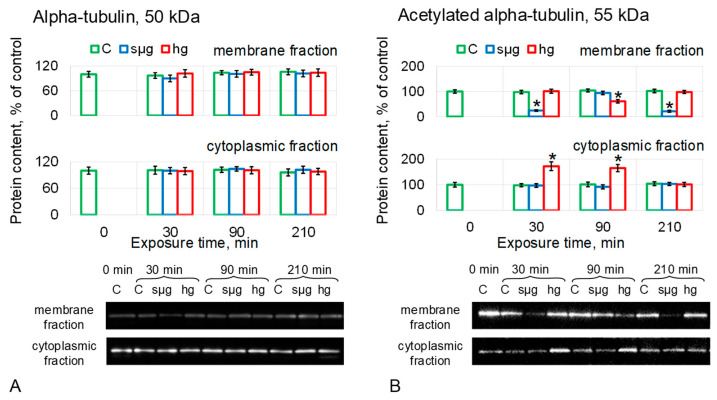
Relative protein content (microtubules proteins) in the membrane and cytoplasmic fractions of *Drosophila melanogaster* oocytes during exposure to simulated micro- and hypergravity. As described above: C, control (green); sµg, simulated microgravity (blue); and hg, hypergravity at the 2 g level (red). Each panel presents typical Western blot (for clarity) and histograms of changes in the content of proteins in the membrane and cytoplasmic fractions as a percentage of the corresponding control at each time point. * *p* < 0.05 in comparison with the corresponding control group. (**A**) Alpha-tubulin, aTub. (**B**) Acetylated alpha-tubulin, acet-aTub, marked stable microtubules.

The relative content of aTub did not change in MF and CF in either simulated microgravity or hypergravity (Figure 4A).

The relative content of acetylated alpha-tubulin in the simulated microgravity group was reduced in MF after 30 and 210 min of exposure by 76% (*p* < 0.05) and 79% (*p* < 0.05), respectively, although after 90 min, it did not differ from the control. There were no changes in the CF of the sµg group (Figure 4B). In the hg group, the content of acet-aTub in MF was reduced by 39% (*p* < 0.05) after 90 min of exposure, although in CF, it exceeded the control level after 30 and 90 min of exposure by 72% (*p* < 0.05) and 65% (*p* < 0.05), respectively (Figure 4B).

The recovery of acetylated alpha-tubulin after 210 min of hypergravity correlates with the data obtained with colchicine on the need for stable microtubules to restore stiffness. The reduced content of acetylated tubulin after 210 min of simulated weightlessness correlates with the non-recovery of oocyte stiffness in this case.

**Figure 5:**
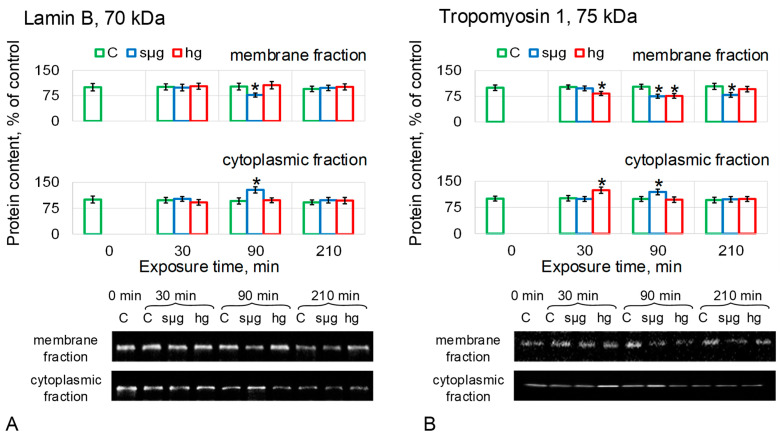
Relative protein content (intermediate filament proteins) in the membrane and cytoplasmic fractions of *Drosophila melanogaster* oocytes during exposure in simulated micro- and hypergravity. As described above: C, control (green); sµg, simulated microgravity (blue); and hg, hypergravity at the 2 g level (red). Each panel presents typical Western blot (for clarity) and histograms of changes in the content of proteins in the membrane and cytoplasmic fractions as a percentage of the corresponding control at each time point. * *p* < 0.05 in comparison with the corresponding control group. (**A**) Lamin B, LamB, formed nuclear intermediate filaments. (**B**) Tropomyosin, Tm1: one of its isoforms; Tm1-I/C, marks cytoplasmic intermediate filaments.

The relative content of LamB in the sµg group decreased in MF after 90 min of exposure by 23% (*p* < 0.05) and at the same time increased in CF by 28% (*p* < 0.05). There were no changes in the relative content of LamB in MF and CF of the hg group (Figure 5A).

The relative content of Tm1 in the MF of the sµg group decreased by 25% (*p* < 0.05) after 90 min of exposure and remained below the control by 21% (*p* < 0.05) after 210 min (Figure 5B). In the sµg CF group, the Tm1 content increased by 19% (*p* < 0.05) after 90 min but decreased to the control level after 210 min. The decrease in the relative content of Tm1 in the MF of oocytes after exposure to hypergravity was 17% after 30 min (*p* < 0.05) and 24% after 90 min (*p* < 0.05), but after 210 min, it was restored to the control level. At the same time, after 30 min in the CF group hg, the content of Tm1 was higher than the control by 24% (*p* < 0.05); after 90 and 210 min, it did not differ from the control (Figure 5B). In other words, the dynamics of changes in Tm1 in the membrane fraction correlate with the dynamics of changes in the stiffness of *Drosophila melanogaster* oocytes in simulated microgravity and hypergravity.

### 3.4. Oocyte’ Cellular Respiration

To assess the functional state of *Drosophila melanogaster* oocytes, the oxygen uptake rate was used.

The rate of oxygen uptake by oocytes in the control group did not change during the experiment from the beginning (0 min) to 210 min.

**Figure 6:**
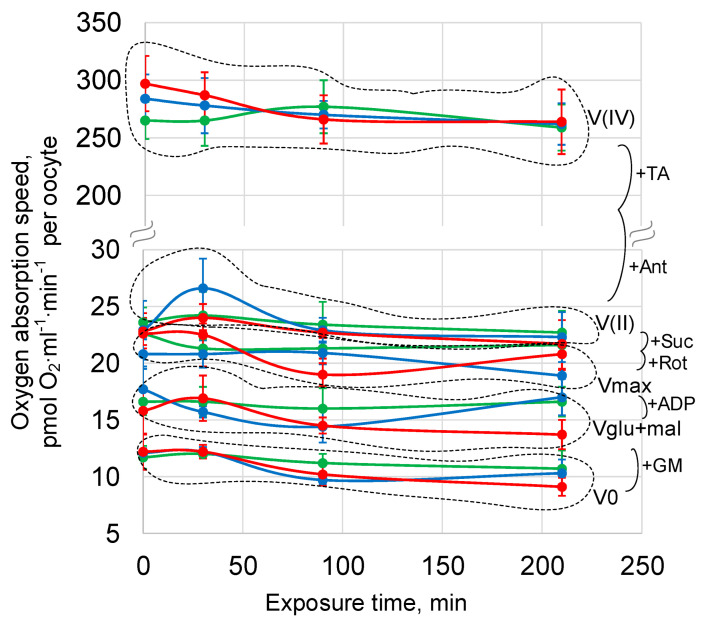
Cellular respiration of *Drosophila melanogaster* oocytes during exposure to simulated micro- and hypergravity. As described above: C, control (green); sµg, simulated microgravity (blue); and hg, hypergravity at the 2 g level (red). V0, basal oxygen absorption. GM, 10 mM glutamate + 5 mM malate. Vglu + mal, oxygen absorption after supplement GM. Vmax, maximal respiratory speed after supplement 2 mM ADP. Rot, 0.5 µM rotenone, inhibitor of the complex I respiratory chain. Suc—10 mM succinate—substrate of the complex II respiratory chain. V (II), respiratory speed after Rot implementation and Suc addition. Ant, 5µM antimycin A, inhibitor of the complex III respiratory chain. TA—0.5 mM TMPD + 2 mM ascorbate—artificial substrates of the complex IV respiratory chain. V (IV), respiratory speed after Ant implementation and TA addition. The dashed line separates groups of estimated parameters.

Exposure of oocytes under simulated micro- and hypergravity for up to 210 min did not lead to a change in the oxygen uptake rate in any link of the respiratory chain: the basal rate V0, the rate of absorption with the addition of substrates of the first complex of the respiratory chain Vglu + mal, the maximum respiratory rate Vmax, and also V (II) and V (IV) (reflecting the functioning of the respiratory chain, starting from complex II and IV, respectively) did not differ from the corresponding control values (Figure 6, Table S2).

## 4. Discussion

In this work, *Drosophila melanogaster* oocytes were used as a convenient object for studying the mechanism of reception of changes in external mechanical stress due to the absence of the need for incubation in liquid, which makes it possible to avoid fluid shift and additional dynamic control. Since the very first events of mechanoreception aroused the main interest, the first exposure point under changed mechanical conditions was chosen to be ultrashort—30 min. The basis for this choice was a number of experimental data indicating that a change in the shape of cells is observed almost immediately under weightless conditions [3], and after 30 min, changes in the structure of the cortical cytoskeleton in glial cells occur [5].

Linear dimensions and the area of the maximum frontal section of oocytes were used as markers for the onset of cell deformation. After 30 min, this area decreases in simulated microgravity and increases in hypergravity (Table 2, Figure 1), which, assuming a constant volume, indicates that the cells become more spherical or flatten, respectively. In other words, there is a different deformation under the conditions of simulated micro- and hypergravity.

Thus, one could expect a change in the structure of the cortical cytoskeleton of oocytes already at the early stages of exposure. Cell stiffness, which is determined by the cytoskeleton, has been used to integrally assess the mechanical structure [19,30,31,32,33]. The measurements made by atomic force microscopy showed that under conditions of simulated microgravity, the stiffness decreased after 90 min and under conditions of 2 g after 30 min, which may indicate a change in the structure of the cytoskeleton (Figure 2A). However, in the case of simulated microgravity, after 210 min, the stiffness remained reduced, and in the case of hypergravity, it was restored to the level of control.

It is interesting that we observed similar dynamics in the early stages in skeletal muscle cells and cardiomyocytes: during antiorthostatic suspension of rats in the earliest periods, stiffness decreases both in m. soleus cells and in cardiomyocytes, although in one case this is a decrease in external mechanical stress, and in another, an increase. Further in dynamics, in the case of m. soleus stiffness remains reduced and then decreases even more. In the case of cardiomyocytes, the cell stiffness first recovers to the control level and then exceeds [34]. In addition, our data on the stiffness of oocytes collected within 12 h after the space flight of the virgin flies *Drosophila melanogaster* show the same (by approximately 15%, *p* < 0.05) decrease in stiffness [19].

For mammalian cells, it has been shown that a decrease in stiffness is most often associated with disrupting microfilaments [32] and is often accompanied by a decrease in the content of beta-, gamma-actin, and actin-binding proteins under simulated microgravity and hypergravity [19,34,35].

Therefore, to elucidate the cause of the decrease in stiffness, studies were carried out using disrupting agents and calyculin A, as well as the content of cytoskeletal proteins in the membrane and cytoplasmic fractions separately.

Surprisingly, pre-incubation with disrupting microfilaments of cytochalasin B did not result in a change in stiffness relative to the non-incubation group and did not affect the dynamics of stiffness change under simulated micro- and hypergravity conditions (Figure 2B). Also, there were no changes in the actin content in the membrane protein fraction, which determines the structure of the cortical cytoskeleton and cell stiffness, in the sµg and hg groups (Figure 3A).

Pre-incubation with disrupted microtubule colchicine did not change the onset time and level of stiffness reduction but prevented its recovery in the hg group after 210 min (Figure 2C). This may indicate that microtubules are necessary for the restoration of the cytoskeleton structure, but this component does not determine the change of the mechanical characteristics of *Drosophila melanogaster* oocytes under simulated micro- and hypergravity. This assumption is supported by data on the content of alpha-tubulin and its acetylated form as a marker of stable microtubules in the membrane fraction of proteins (Figure 4A,B): after 210 min in the sµg group, the content of acetylated alpha-tubulin was lower than the control, as well as stiffness. In the hg group, the acet-aTub content recovered to the control level, as did the stiffness. However, the decrease in acet-aTub content in the sµg group occurred earlier than the decrease in stiffness, suggesting the role of another cytoskeletal component in the formation of stiffness.

It seems interesting that pre-incubation with acrylamide, which specifically disrupts intermediate filaments without affecting other structures of the cytoskeleton [36], leads to a decrease in stiffness in the control group to the same level as when exposed to simulated micro- and hypergravity. Moreover, the stiffness does not change in the dynamics of exposure under simulated micro- and hypergravity and remains as low as at the beginning of the exposure (Figure 2D). This indicates that intermediate filaments are at least involved in the observed decrease in *Drosophila melanogaster* oocyte stiffness under changes in external mechanical stress. However, it was previously believed that *Drosophila* lack cytoplasmic intermediate filaments [37]. Therefore, the content of the known component of intermediate filaments Lamin B, which is a nuclear lamina protein, was first determined, and naturally, no correlation was found with cell stiffness (Figure 5A); only a decrease in the membrane fraction was noted after 90 min of exposure to simulated microgravity and a corresponding increase in the cytoplasmic fraction. There were no changes in the hg group.

However, recent data indicate that Drosophila have cytoplasmic intermediate filaments formed by the Tm1-I/C protein [38,39]. Indeed, the dynamics of changes in the content of Tm1 in the membrane fraction of proteins in the sµg and hg groups (Figure 5B) correlate with changes in oocyte stiffness: it decreases after 90 min in the sµg group and remains reduced up to 210 min; in the hg group it decreases after 30 min, remains reduced after 90 min, but recovers to control levels after 210 min. Interestingly, the primary decrease in the membrane fraction in both groups was associated with the accumulation of Tm1 in the cytoplasmic fraction, and further dynamics of the content in the cytoplasmic fraction seemed to be determined by mechanotransduction signaling pathways and the synthesis-proteolysis balance. In addition, it should be noted that the antibodies used can bind to epitopes common to all Tm1 isoforms; therefore, the dynamics of the content in the cytoplasm may reflect a change not only in Tm1-I/C but also in the isoform associated with intracellular transport.

Intermediate filaments formed by Tm1-I/C are required for collective border cell migration, in epithelial cells for proper cytoarchitecture, and in the germline for the formation of germ plasm [38]. In addition, in the maturing oocyte (stage 7), the filaments formed by Tm1-I/C do not overlap with F-actin but are colocalized with alpha-tubulin [38]. The latter correlates with the non-restoration of stiffness to control with disassembled microtubules in the simulated microgravity group.

Thus, the stiffness of *Drosophila melanogaster* oocytes was changed, possibly as a result of changes in the structure of intermediate filaments and in general due to the integrative rearrangement of the cortical cytoskeleton. The decrease in stiffness in the sµg and hg groups in both cases was associated with the destruction of intermediate filaments, the restoration of which requires microtubules. However, different stiffness dynamics indicate the triggering of different signaling pathways in the case of simulated microgravity and hypergravity. However, the question remains as to how a single cell perceives differences in changes in external mechanical stress.

Previously, we suggested that an increase or decrease in external mechanical stress leads to deformation of compression or stretching of the cortical cytoskeleton, leading to the dissociation of various proteins from it and the subsequent triggering of various signaling pathways [1,2]. This was confirmed in this study by a decrease and increase in the cross-sectional area of oocytes under simulated micro- and hypergravity, respectively (Figure 1). For mammalian cells, whose stiffness is determined by microfilaments, such candidates may be actin-binding proteins, particularly alpha-actinin isoforms [34].

Although microfilaments do not appear to determine the changes in cortical cytoskeletal stiffness in *Drosophila melanogaster* oocytes in vivo under simulated micro- and hypergravity, pre-incubation with calyculin A resulted in its increase (Figure 2E). Calyculin A stimulates the condensation of cortical actin, leading to the appearance of additional stress fibrils [22,23]. This increase in stiffness does not prevent its reduction when exposed to simulated microgravity and hypergravity compared to the corresponding control; however, the level of reduction becomes not so significant. These data suggest that the mechanosensitivity of *Drosophila melanogaster* oocytes may involve proteins that interact with both intermediate filaments and microfilaments, for example, fascin [40], alpha-actinin [41], and spectrin [42].

The Singed protein, which is a homologue of fascin, a protein that binds microfilaments into longitudinal bundles, decreased in the membrane fraction after 30 min under simulated microgravity and migrated to the cytoplasmic fraction, where its content increased after 90 min of exposure (Figure 3B). At the same time, in hypergravity, its content in the membrane fraction remains intact, and in the cytoplasmic fraction, it increases only after 210 min. Alpha-actinin, which organizes microfilaments into another type of structure—loose networks—as well as spectrin, migrate from the membrane fraction to the cytoplasmic one after 30 min under hypergravity conditions (Figure 3C,D), but remain intact under microgravity conditions. Thus, in response to simulated microgravity, proteins organizing longitudinal bundles of filaments migrate from the cortical cytoskeleton of *Drosophila melanogaster* oocytes, and proteins organizing filament networks migrate in response to hypergravity. Such a difference can explain the mechanosensitivity and the formation of a different adaptive pattern of the structure under simulated microgravity compared to hypergravity.

Changes in the structure of the cytoskeleton can affect the functional status of oocytes. The migration of mitochondria and their position in cells depend both on microtubules [4] and on the actin network [43,44], the latter playing a role both in maintaining the mitochondrial membrane potential and in oxidation–phosphorylation coupling [16]. In addition, it was shown in mammalian muscle cells that desmin, one of the proteins of cytoplasmic intermediate filaments, determines the localization of mitochondria [45] and, in its absence, structural and functional changes in mitochondria were noted [46,47], in particular, a decrease in the rate of oxygen consumption [48]. Therefore, taking into account the data on the structure of the cytoskeleton, we expected to detect a change in the rate of oxygen uptake by oocytes. However, the rate of cellular respiration did not change in either simulated microgravity or hypergravity (Figure 6). It can be assumed that such a short exposure time (maximum 210 min) and transient (during this period of exposure) changes in the structure of the cytoskeleton do not lead to a violation of the rate of ATP synthesis in the mitochondria, and the observed changes in the structure do not depend on the metabolic status of cells.

## 5. Conclusions

**Figure 7:**
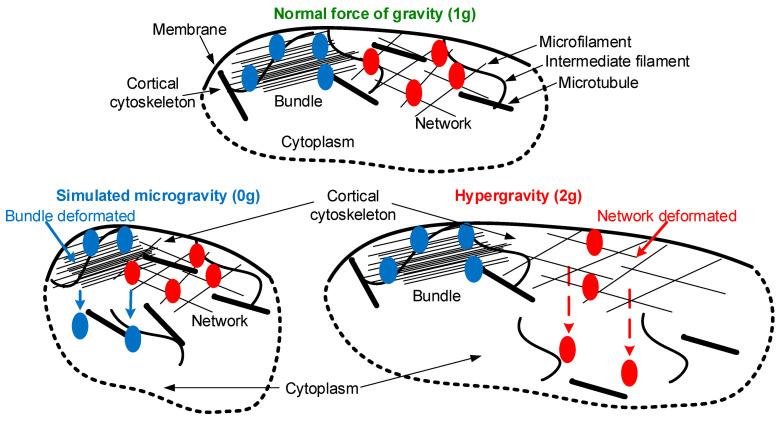
Possible scheme of primary acts of mechanoreception. The cell membrane and the interacting filaments, that form the cortical cytoskeleton, are shown in black: microfilaments, intermediate filaments, and microtubules. Blue indicates proteins that organize longitudinal bundles, in particular singed, which migrates into the cytoplasm from the cortical cytoskeleton under simulated microgravity. Red indicates proteins that organize networks; in particular alpha-actinin and spectrin migrate into the cytoplasm under hypergravity conditions. When the external mechanical stress changes from 1 g to 0 g or 2 g, in accordance with Newton’s third law, the internal stress changes and deformation occurs, resulting in compression or stretching. It leads to dissociation from the cortical cytoskeleton of various proteins (blue or red, respectively). Detailed explanations of the sequence of events are provided in the text (Section 5).

Summarizing the obtained results, we propose the following sequence of events (Figure 7), modifying the scheme proposed earlier in the review [2]. Normally (under 1 g conditions), the stiffness of the cortical cytoskeleton of *Drosophila melanogaster* oocytes is determined by the interaction of different components of the cytoskeleton, microfilaments, intermediate filaments, and microtubules. In accordance with Newton’s third law, a change in external mechanical stress leads to an oppositely directed internal stress and, accordingly, to cell deformation. Under conditions of simulated micro- and hypergravity, various proteins migrate from it into the cytoplasm due to various deformations of the cortical cytoskeleton. In the case of simulated microgravity, the singed protein migrates, binding microfilaments into longitudinal bundles. In the case of hypergravity, the proteins alpha-actinin and spectrin, which organize filaments in a network, migrate away from the cortical cytoskeleton. In other words, in the case of transition to microgravity conditions, deformation of the longitudinal bundles occurs, and in the case of transition to hypergravity conditions, the deformation of networks occurs. Further, the dissociation of binding proteins leads to a decrease in the number of intermediate filaments, the integrative rearrangement of the cortical cytoskeleton, and a decrease in stiffness. Stable microtubules are required to restore the structure of intermediate filaments in the cortical layer, which emphasizes the role of the cortical cytoskeleton as a whole. The return of stiffness to the control level under hypergravity conditions and the non-return under simulated microgravity conditions demonstrate different strategies for the formation of an adaptive structure pattern, even at a short exposure—maximum of 210 min in this study. Structural changes apparently precede metabolic ones, since one of the key parameters, cellular respiration, does not change during the entire exposure either in simulated microgravity or in hypergravity. Thus, the results obtained support the hypothesis of the role of the cortical cytoskeleton as a mechanosensor, and the migration of proteins from it that form various structures (bundles and networks) with a decrease and increase in external mechanical stress may be the first act of mechanoreception.

## 6. Limitations of the Study

*Drosophila melanogaster* oocytes are an extremely convenient object for this kind of research, primarily due to the possibility of exposure to a humid environment rather than a liquid. However, their large cell size limits the use of confocal microscopy. Our attempts to visualize changes in the structure of the cortical cytoskeleton were not successful; we failed to obtain high-quality images of individual filaments. The absence of such images is a significant limitation of this study, just as the estimation of the area of the maximum frontal section of oocytes by light microscopy is not the best marker.

This study was able to identify proteins showing different responses to simulated microgravity and hypergravity. However, the spectrum of such proteins can be much wider, and their identification requires, first of all, screening studies, for example, proteomic analysis using mass spectrometry. Finally, commercially available anti-Tm1 antibodies bind to an epitope common to all isoforms, including Tm1-I/C, which imposes restrictions on the interpretation of the obtained data, especially for the cytoplasmic fraction of proteins.

## Figures and Tables

**Table 1:** Primary antibodies.

Protein	Manufacturer with Catalog Number, Dilution
Actb (beta-actin, 42 kDa)	#ab227387, Abcam, Cambridge, UK, 1:5000
Sn (singed, 57 kDa)	Sn 7C was deposited to the DSHB by Cooley, L., DSHB Hybridoma Product, Iowa City, IA, USA, 0.5 µg/mL
Actn (alpha-actinin, 103 kDa)	#ab50599, Abcam, Cambridge, UK, 1 µg/mL
AlphaSpec (spectrin alpha, 234 kDa)	3A9 was deposited to the DSHB by Branton, D./Dubreuil, R., DSHB Hybridoma Product, Iowa City, IA, USA, 0.5 µg/mL
aTub (alpha-tubulin, 50 kDa)	#ab52866, Abcam, Cambridge, UK, 1:10,000
acet-aTub (acetylated alpha-tubulin, 55 kDa)	#sc-23950, Santa Cruz Biotechnology, Inc., Santa Cruz, CA, USA, 1:500
LamB (LaminB, 71 kDa)	ADL40 was deposited to the DSHB by Fisher, P. A., DSHB Hybridoma Product, Iowa City, IA, USA, 0.5 µg/mL
Tm1 (tropomyosin, 75 kDa)	BB5/37.1 was deposited to the DSHB by Bullard, Belinda, DSHB Hybridoma Product, Iowa City, IA, USA, 0.5 µg/mL

**Table 2:** Linear sizes (minor and major axes) of *Drosophila melanogaster* oocytes under simulated micro- and hypergravity.

Group		0 min	30 min	90 min	210 min
C	1—L_min_, µm	172.4 ± 1.1	173.2 ± 0.9	173.0 ± 0.8	173.4 ± 0.8
	2—L_max_, µm	525.0 ± 2.1	525.0 ± 2.2	524.7 ± 1.5	525.5 ± 3.0

sµg	1—L_min_, µm		175.4 ± 1.0 ^&^	173.2 ± 0.9	170.9 ± 0.8 *
	2—L_max_, µm		517.2 ± 2.7 *	515.7 ± 1.5 *	517.4 ± 2.6 *

hg	1—L_min_, µm		178.2 ± 0.7 *	178.2 ± 0.7 *	172.6 ± 0.7
	2—L_max_, µm		530.7 ± 1.8 *	520.4 ± 1.8 ^&^	518.1 ± 1.9 *

C, control. sµg, simulated microgravity. hg, hypergravity at the 2 g level. 1, L_min_: minimal linear size of the *Drosophila melanogaster* oocytes (purple line 1 on the right panel Figure 1). 2, L_max_: maximal linear size of the *Drosophila melanogaster* oocytes (purple line 2 on the right panel Figure 1). * *p* < 0.05 in comparison with the according control group without any agents, and ^&^ *p* < 0.1 in comparison with the according control group without any agents.

## Data Availability

All data generated or analyzed during this study are included in this article and Supplementary Materials.

## Supplementary Materials

The following supporting information can be downloaded at: https://www.mdpi.com/article/10.3390/cells12141819/s1, Table S1: The stiffness (in pN/nm) of the *Drosophila melanogaster’* oocytes under simulated micro- and hypergravity without or with addition agents; Table S2: The cellular respiration (in pmol O_2_∙mL^−1^∙min^−1^∙oocyte^−1^) of the *Drosophila melanogaster’* oocytes under simulated micro- and hypergravity.

## References

[1] Ogneva I.V. (2013). Cell mechanosensitivity: Mechanical properties and interaction with gravitational field. Biomed. Res. Int..

[2] Ogneva I.V. (2022). Single Cell in a Gravity Field. Life.

[3] Thiel C.S., Tauber S., Lauber B., Polzer J., Seebacher C., Uhl R., Neelam S., Zhang Y., Levine H., Ullrich O. (2019). Rapid Morphological and Cytoskeletal Response to Microgravity in Human Primary Macrophages. Int. J. Mol. Sci..

[4] Schatten H., Lewis M.L., Chakrabarti A. (2001). Spaceflight and clinorotation cause cytoskeleton and mitochondria changes and increases in apoptosis in cultured cells. Acta Astronaut..

[5] Uva B.M., Masini M.A., Sturla M., Prato P., Passalacqua M., Giuliani M., Tagliafierro G., Strollo F. (2002). Clinorotation-induced weightlessness influences the cytoskeleton of glial cells in culture. Brain Res..

[6] Gaboyard S., Blanchard M.P., Travo C., Viso M., Sans A., Lehouelleur J. (2002). Weightlessness affects cytoskeleton of rat utricular hair cells during maturation in vitro. Neuroreport.

[7] Kacena M.A., Todd P., Landis W.J. (2003). Osteoblasts subjected to spaceflight and simulated space shuttle launch conditions. Vitr. Cell Dev. Biol. Anim..

[8] Crawford-Young S.J. (2006). Effects of microgravity on cell cytoskeleton and embryogenesis. Int. J. Dev. Biol..

[9] Corydon T.J., Kopp S., Wehland M., Braun M., Schütte A., Mayer T., Hülsing T., Oltmann H., Schmitz B., Hemmersbach R. (2016). Alterations of the cytoskeleton in human cells in space proved by life-cell imaging. Sci. Rep..

[10] Ogneva I.V., Zhdankina Y.S., Kotov O.V. (2022). Sperm of Fruit Fly *Drosophila melanogaster* under Space Flight. Int. J. Mol. Sci..

[11] Thiel C.S., de Zélicourt D., Tauber S., Adrian A., Franz M., Simmet D.M., Schoppmann K., Hauschild S., Krammer S., Christen M. (2017). Rapid adaptation to microgravity in mammalian macrophage cells. Sci. Rep..

[12] Desplanches D., Mayet M.H., Sempore B., Frutoso J., Flandrois R. (1987). Effect of spontaneous recovery or retraining after hindlimb suspension on aerobic capacity. J. Appl. Physiol. (1985).

[13] Bigard A.X., Boehm E., Veksler V., Mateo P., Anflous K., Ventura-Clapier R. (1998). Muscle unloading induces slow to fast transitions in myofibrillar but not mitochondrial properties. Relevance to skeletal muscle abnormalities in heart failure. J. Mol. Cell. Cardiol..

[14] Ogneva I.V., Usik M.A. (2021). Mitochondrial Respiration in *Drosophila* Ovaries after a Full Cycle of Oogenesis under Simulated Microgravity. Curr. Issues Mol. Biol..

[15] Bartolak-Suki E., Imsirovic J., Nishibori Y., Krishnan R., Suki B. (2017). Regulation of Mitochondrial Structure and Dynamics by the Cytoskeleton and Mechanical Factors. Int. J. Mol. Sci..

[16] Xie X., Venit T., Drou N., Percipalle P. (2018). In Mitochondria beta-Actin Regulates mtDNA Transcription and Is Required for Mitochondrial Quality Control. iScience.

[17] Ingber D.E., Wang N., Stamenovic D. (2014). Tensegrity, cellular biophysics, and the mechanics of living systems. Rep. Prog. Phys..

[18] Ogneva I.V., Biryukov N.S. (2016). Lecithin Prevents Cortical Cytoskeleton Reorganization in Rat Soleus Muscle Fibers under Short-Term Gravitational Disuse. PLoS ONE.

[19] Ogneva I.V., Golubkova M.A., Biryukov N.S., Kotov O.V. (2022). *Drosophila melanogaster* Oocytes after Space Flight: The Early Period of Adaptation to the Force of Gravity. Cells.

[20] Tran S.L., Welte M.A. (2010). In-vivo centrifugation of *Drosophila* embryos. J. Vis. Exp..

[21] Ofek G., Wiltz D.C., Athanasiou K.A. (2009). Contribution of the cytoskeleton to the compressive properties and recovery behavior of single cells. Biophys. J..

[22] Cybulsky A.V., Takano T., Papillon J., Khadir A., Bijian K., Le Berre L. (2004). The actin cytoskeleton facilitates complement-mediated activation of cytosolic phospholipase A2. Am. J. Physiol. Renal. Physiol..

[23] Phan T.K.T., Do T.L., Tachibana K., Kihara T. (2022). Alpha-mangostin dephosphorylates ERM to induce adhesion and decrease surface stiffness in KG-1 cells. Hum. Cell.

[24] Marco R., Laván D.A., van Loon J.J., Leandro L.J., Larkin O.J., Dijkstra C., Anthony P., Villa A., Davey M.R., Lowe K.C. (2007). *Drosophila melanogaster*, a model system for comparative studies on the responses to real and simulated microgravity. J. Gravit. Physiol..

[25] Furukawa T., Tanimoto K., Fukazawa T., Imura T., Kawahara Y., Yuge L. (2018). Simulated microgravity attenuates myogenic differentiation via epigenetic regulations. NPJ Microgravity.

[26] Otsuka T., Imura T., Nakagawa K., Shrestha L., Takahashi S., Kawahara Y., Sueda T., Kurisu K., Yuge L. (2018). Simulated Microgravity Culture Enhances the Neuroprotective Effects of Human Cranial Bone-Derived Mesenchymal Stem Cells in Traumatic Brain Injury. Stem Cells Dev..

[27] Imura T., Nakagawa K., Kawahara Y., Yuge L. (2018). Stem Cell Culture in Microgravity and Its Application in Cell-Based Therapy. Stem Cells Dev..

[28] Vitorino R., Ferreira R., Neuparth M., Guedes S., Williams J., Tomer K.B., Domingues P.M., Appell H.J., Duarte J.A., Amado F.M. (2007). Subcellular proteomics of mice gastrocnemius and soleus muscles. Anal. Biochem..

[29] Kuznetsov A.V., Veksler V., Gellerich F.N., Saks V., Margreiter R., Kunz W.S. (2008). Analysis of mitochondrial function in situ in permeabilized muscle fibers, tissues and cells. Nat. Protoc..

[30] Ogneva I.V. (2010). Transversal stiffness of fibers and desmin content in leg muscles of rats under gravitational unloading of various durations. J. Appl. Physiol..

[31] Mathur A.B., Collinsworth A.M., Reichert W.M., Kraus W.E., Truskey G.A. (2001). Endothelial, cardiac muscle and skeletal muscle exhibit different viscous and elastic properties as determined by atomic force microscopy. J. Biomech..

[32] Costa K.D. (2006). Imaging and probing cell mechanical properties with the atomic force microscope. Methods Mol. Biol..

[33] Cai X., Gao S., Cai J., Wu Y., Deng H. (2009). Artesunate induced morphological and mechanical changes of Jurkat cell studied by AFM. Scanning.

[34] Collinsworth A.M., Zhang S., Kraus W.E., Truskey G.A. (2002). Apparent elastic modulus and hysteresis of skeletal muscle cells throughout differentiation. Am. J. Physiol. Cell Physiol..

[35] Ogneva I.V., Biryukov N.S., Leinsoo T.A., Larina I.M. (2014). Possible role of non-muscle alpha-actinins in muscle cell mechanosensitivity. PLoS ONE.

[36] Eckert B.S. (1986). Alteration of the distribution of intermediate filaments in PtK1 cells by acrylamide. II: Effect on the organization of cytoplasmic organelles. Cell Motil. Cytoskelet..

[37] Erber A., Riemer D., Bovenschulte M., Weber K. (1998). Molecular phylogeny of metazoan intermediate filament proteins. J. Mol. Evol..

[38] Cho A., Kato M., Whitwam T., Kim J.H., Montell D.J. (2016). An Atypical Tropomyosin in *Drosophila* with Intermediate Filament-like Properties. Cell Rep..

[39] Sysoev V.O., Kato M., Sutherland L., Hu R., McKnight S.L., Murray D.T. (2020). Dynamic structural order of a low-complexity domain facilitates assembly of intermediate filaments. Proc. Natl. Acad. Sci. USA.

[40] Mondal S., Dirks P., Rutka J.T. (2010). Immunolocalization of fascin, an actin-bundling protein and glial fibrillary acidic protein in human astrocytoma cells. Brain Pathol..

[41] Bolanos S.H., Zamora D.O., García D.M., Koke J.R. (1998). An alpha-actinin isoform which may cross-link intermediate filaments and microfilaments. Cytobios.

[42] Yang Z., Mattingly B.C., Hall D.H., Ackley B.D., Buechner M. (2020). Terminal web and vesicle trafficking proteins mediate nematode single-cell tubulogenesis. J. Cell Biol..

[43] Boldogh I.R., Pon L.A. (2006). Interactions of mitochondria with the actin cytoskeleton. Biochim. Biophys. Acta.

[44] Senning E.N., Marcus A.H. (2010). Actin polymerization driven mitochondrial transport in mating S. cerevisiae. Proc. Natl. Acad. Sci. USA.

[45] Capetanaki Y., Bloch R.J., Kouloumenta A., Mavroidis M., Psarras S. (2007). Muscle intermediate filaments and their links to membranes and membranous organelles. Exp. Cell Res..

[46] Rappaport L., Oliviero P., Samuel J.L. (1998). Cytoskeleton and mitochondrial morphology and function. Mol. Cell Biochem..

[47] Capetanaki Y., Milner D.J. (1998). Desmin cytoskeleton in muscle integrity and function. Subcell. Biochem..

[48] Milner D.J., Mavroidis M., Weisleder N., Capetanaki Y. (2000). Desmin cytoskeleton linked to muscle mitochondrial distribution and respiratory function. J. Cell Biol..

