# Supplementary material for: The Mechanoreception in Drosophila melanogaster Oocyte under Modeling Micro- and Hypergravity
Source: Cells. 2023 Jul 10;12(14):1819. doi: 10.3390/cells12141819 (PMC10377865; doi:10.3390/cells12141819)
Supplement: Supplementary file 1 [file cells-12-01819-s001.zip › cells-2472608-supplementary.pdf]

The mechanoreception in *Drosophila melanogaster* oocyte under modeling micro- and hypergravity

Irina V. Ogneva [1,2]*
[1] Cell Biophysics Laboratory, State Scientific Center of the Russian Federation Institute of Biomedical Problems of the Russian Academy of Sciences, 76a, Khoroshevskoyoe shosse, 123007 Moscow, Russia;
[2] Medical and biological physics department, I. M. Sechenov First Moscow State Medical University, 8-2 Trubetskaya Street, 119991, Moscow, Russia;
*Correspondence: iogneva@yandex.ru, iogneva@imbp.ru; Tel.: +7(499)195-63-98

Table S1. The stiffness (in pN/nm) of the *Drosophila melanogaster*' oocytes under simulated micro- and hypergravity without or with addition agents.

Group and conditions of pre-incubation / Exposure time		0 min	30 min	90 min	210 min
No pre-incubation	C	30.7 ±1.0	30.8 ±1.4	31.2 ±1.2	29.6 ±0.7
	sμg	30.4 ±1.4	30.6 ±1.3	25.5 ±1.2*↓	24.8 ±1.2*↓
	hg	31.4 ±1.0	26.7 ±1.8*↓	27.0 ±2.4*↓	29.5 ±1.4
Pre-incubation in 0.65% NaCl without any agents 3 hours, RT	C	31.0 ±1.4	31.5 ±0.9	31.3 ±1.8	30.6 ±1.6
	sμg	30.8 ±1.5	31.1 ±1.6	25.8 ±1.8*↓	26.2 ±1.4*↓
	hg	30.6 ±1.1	26.7 ±1.7*↓	26.3 ±1.4*↓	31.2 ±1.1
Pre-incubation in 0.65% NaCl + 2 μM cytochalasine B 3 hours, RT	C	30.4 ±1.2	31.0 ±1.6	30.5 ±2.1	30.0 ±1.1
	sμg	30.9 ±1.4	31.5 ±1.3	26.0 ±1.2*↓	26.3 ±1.8*↓
	hg	30.4 ±1.2	26.7 ±2.1*↓	26.3 ±1.4*↓	30.8 ±1.6
Pre-incubation in 0.65% NaCl + 10 μM colchicine 3 hours, RT	C	31.1 ±2.1	31.5 ±1.1	30.4 ±1.5	30.4 ±1.0
	sμg	31.1 ±1.4	30.4 ±1.2	25.8 ±0.7*↓	26.0 ±1.4*↓
	hg	31.5 ±1.7	26.6 ±2.0*↓	26.0 ±1.2*↓	25.8 ±0.9*↓
Pre-incubation in 0.65% NaCl + 40mM acrylamide 3 hours, RT	C	26.4 ±0.8*↓	27.0 ±1.4*↓	26.6 ±1.4*↓	25.8 ±1.2*↓
	sμg	26.2 ±0.9*↓	27.1 ±1.2*↓	25.9 ±0.8*↓	26.7 ±1.1*↓
	hg	26.7 ±1.2*↓	25.9 ±1.2*↓	26.2 ±1.2*↓	25.3 ±1.2*↓
Pre-incubation in 0.65% NaCl + 100nM calyculin A 3 hours, RT	C	36.1 ±1.8*↑	35.7 ±1.6*↑	35.6 ±1.0*↑	35.3 ±1.3*↑
	sμg	34.7 ±1.6*↑	35.9 ±1.5*↑	30.1 ±1.2	30.2 ±1.7
	hg	35.3 ±1.4*↑	28.2 ±2.0[&]↓	30.5 ±1.1	30.4 ±2.0

C – control, sμg – simulated microgravity, hg – hypergravity at the 2g level. RT – room temperature. * – $p < 0.05$ in comparison with the according control group without any agents, [&] – $p < 0.1$ in comparison with the according control group without any agents. ↑ – increase relative to the reference value. ↓ – decrease relative to the reference value.

Table S2. The cellular respiration (in pmol $O_2 \cdot ml^{-1} \cdot min^{-1} \cdot oocyte^{-1}$) of the *Drosophila melanogaster*' oocytes under simulated micro- and hypergravity.

Group \ Exposure time		0 min	30 min	90 min	210 min
V0	C	11.7 ±1.0	12.0 ±0.4	11.2 ±0.8	10.7 ±1.6
	sμg	12.1 ±0.4	12.2 ±0.4	9.7 ±0.4	10.3 ±1.2
	hg	12.2 ±1.6	12.2 ±0.6	10.2 ±1.0	9.1 ±0.8
Vglu+mal	C	16.6 ±1.2	16.6 ±1.3	16.0 ±1.8	16.6 ±1.3
	sμg	17.7 ±2.0	15.7 ±0.5	14.4 ±1.4	17.0 ±1.6
	hg	15.8 ±2.1	16.9 ±2.0	14.5 ±0.7	13.7 ±1.3
Vmax	C	22.6 ±1.8	21.3 ±1.6	21.3 ±0.9	21.6 ±2.2
	sμg	20.8 ±1.3	20.8 ±1.2	20.9 ±1.0	18.9 ±1.9
	hg	22.6 ±1.3	22.5 ±1.0	19.0 ±1.0	20.8 ±1.3
V(II)	C	23.6 ±1.3	24.2 ±2.1	23.4 ±2.0	22.7 ±1.9
	sμg	22.9 ±2.6	26.6 ±2.6	22.9 ±1.1	22.3 ±2.2
	hg	22.8 ±1.2	24.0 ±1.2	22.7 ±0.4	21.7 ±1.1
V(IV)	C	265 ±16	265 ±22	277 ±23	259 ±20
	sμg	284 ±21	278 ±24	270 ±12	262 ±18
	hg	297 ±24	287 ±20	266 ±21	264 ±28

C – control, sμg – simulated microgravity, hg – hypergravity at the 2g level. V0 – basal oxygen absorption. Vglu+mal – oxygen absorption after supplement 10mM glutamate+5mM malate (substrates of the complex I respiratory chain). Vmax – maximal oxygen absorption after supplement 2mM ADP. V(II) – oxygen absorption after 0.5 μM rotenone implementation (inhibitor of the complex I respiratory chain) and 10 mM succinate addition (substrate of the complex II respiratory chain). V(IV) – oxygen absorption after 5 μM antimycin A implementation (inhibitor of the complex III respiratory chain) and 0.5 mM TMPD + 2 mM ascorbate addition (artificial substrates of the complex IV respiratory chain).
